# ID 156 - Pegcetacoplana como Opção Terapêutica no Tratamento de Hemoglobinúria Paroxística Noturna: monitoramento de horizonte tecnológico

**DOI:** 10.5327/2237-9622.2025.v34s1.156

**Published:** 2025-11-25

**Authors:** Helder Cassio de Oliveira, Ternize Mariana Guenkka, Kelli Carneiro de Freitas Nakata

**Keywords:** monitoramento de horizonte tecnológico, hemoglobinúria paroxística noturna, doenças raras

## Abstract

**Introdução:**

A hemoglobinúria paroxística noturna (HPN) é um distúrbio raro causado por uma mutação genética nas células-tronco, resultando na ausência de proteínas reguladoras do Sistema de Complemento, o que pode levar à hemólise intravascular, trombose, e insuficiências de órgãos. No Brasil, em 2023, a prevalência de HPN foi de 1 caso para cada 237 mil pessoas atendidas pelo SUS. O Protocolo Clínico da HPN recomenda o transplante de células-tronco hematopoéticas como o único tratamento curativo, além do uso de eculizumabe para bloquear a hemólise e melhorar a qualidade de vida, reduzindo o risco de trombose. A pegcetacoplana liga-se à proteína C3, inibindo a ativação do Sistema de Complemento, regulando a hemólise. Este estudo apresenta o monitoramento de horizonte tecnológico (MHT) da pegcetacoplana para o tratamento da HPN.

**Método:**

O MHT foi realizado em duas etapas: (1) Busca por protocolos clínicos sobre o uso de pegcetacoplana nas plataformas Cortellis e ClinicalTrials, incluindo ensaios clínicos de fase 3, randomizados ou não; (2) Recuperação de estudos nas bases PubMed, Embase, LILACS e Cochrane Library. Duplicatas foram removidas pelo software Rayyan, e dois avaliadores selecionaram os estudos de forma independente. Foram excluídos estudos in vitro, com animais, farmacocinéticos, revisões e análises post hoc.

**Resultados:**

Foram recuperados 480 títulos, com 182 duplicatas. Dez estudos foram selecionados para leitura completa, resultando em quatro estudos clínicos: três de fase 3 (PEGASUS, PRINCE e OLE) e um estudo de mundo real. No estudo PEGASUS, comparando eculizumabe e pegcetacoplana, 24% dos pacientes no grupo pegcetacoplana e 26% no grupo eculizumabe não necessitaram de transfusões nos últimos 12 meses. Entre os que precisaram de quatro ou mais transfusões, 51% estavam no grupo pegcetacoplana e 59% no grupo eculizumabe. A hemoglobina média foi de 8,69g/dL no grupo pegcetacoplana e 8,68g/dL no grupo eculizumabe. Quanto à pontuação FACIT-F (fadiga), os resultados foram 32,2 para pegcetacoplana e 31,6 para eculizumabe.

No estudo PRINCE, com pacientes virgens de inibidores do complemento, 82,9% do grupo-intervenção e 77,8% do grupo-controle precisaram de transfusões. A hemoglobina média foi de 9,4g/dL no grupo-intervenção e 8,7g/dL no controle, com FACIT-F de 36,3 e 37,1, respectivamente. O estudo OLE está em andamento. Já o estudo de mundo real indicou que 72,9% usaram pegcetacoplana devido à hemólise extravascular e transfusões no último ano. No início do tratamento, a hemoglobina média foi de 91g/L, com uma contagem de reticulócitos de 205x109/L, que caiu para 107x109/L após três meses, sugerindo redução na produção de células vermelhas. A pegcetacoplana foi bem tolerada, com eventos adversos como hemólise, fadiga, eritema no local da injeção e infecções. Alguns pacientes, no entanto, apresentaram hemólise intravascular avançada, exigindo uso intensivo da pegcetacoplana.

**Conclusão:**

A pegcetacoplana é o primeiro medicamento que inibe o complemento C3, indicado tanto para pacientes virgens quanto para aqueles que apresentam hemólise residual mesmo com inibidores de C5. As evidências indicam que a pegcetacoplana melhora parâmetros hematológicos, reduzindo a necessidade de transfusões e a fadiga. Em comparação com o eculizumabe, o tratamento com pegcetacoplana tem se mostrado eficaz em manter os níveis de hemoglobina e reduzir a dependência de transfusões, sendo uma opção promissora para uma ampla gama de pacientes com HPN.

